# Expression of TP53 Isoforms p53β or p53γ Enhances Chemosensitivity in TP53^null^ Cell Lines

**DOI:** 10.1371/journal.pone.0056276

**Published:** 2013-02-11

**Authors:** Elisabeth Silden, Sigrun M. Hjelle, Line Wergeland, André Sulen, Vibeke Andresen, Jean-Christophe Bourdon, David R. Micklem, Emmet McCormack, Bjørn Tore Gjertsen

**Affiliations:** 1 Hematology Section, Institute of Medicine, University of Bergen, Bergen, Norway; 2 Inserm-European Associated Laboratory Inserm U858, Department of Surgery and Molecular Oncology, University of Dundee Medical School, Dundee, Scotland, United Kingdom; 3 Institute of Biomedicine, University of Bergen, Bergen, Norway; 4 Hematology Section, Department of Internal Medicine, Haukeland University Hospital, Bergen, Norway; University of Illinois at Chicago, United States of America

## Abstract

The carboxy-terminal truncated p53 alternative spliced isoforms, p53β and p53γ, are expressed at disparate levels in cancer and are suggested to influence treatment response and therapy outcome. However, their functional role in cancer remains to be elucidated. We investigated their individual functionality in the p53^null^ background of cell lines H1299 and SAOS-2 by stable retroviral transduction or transient transfection. Expression status of p53β and p53γ protein was found to correlate with increased response to camptothecin and doxorubicin chemotherapy. Decreased DNA synthesis and clonogenicity in p53β and p53γ congenic H1299 was accompanied by increased p21^(CIP1/WAF1)^, Bax and Mdm2 proteins. Chemotherapy induced p53 isoform degradation, most prominent for p53γ. The proteasome inhibitor bortezomib substantially increased basal p53γ protein level, while the level of p53β protein was unaffected. Treatment with dicoumarol, a putative blocker of the proteasome-related NAD(P)H quinone oxidoreductase NQO1, effectively attenuated basal p53γ protein level in spite of bortezomib treatment. Although *in vitro* proliferation and clonogenicity assays indicated a weak suppressive effect by p53β and p53γ expression, studies of *in vivo* subcutaneous H1299 tumor growth demonstrated a significantly increased growth by expression of either p53 isoforms. This study suggests that p53β and p53γ share functionality in chemosensitizing and tumor growth enhancement but comprise distinct regulation at the protein level.

## Introduction

**Figure 1:**
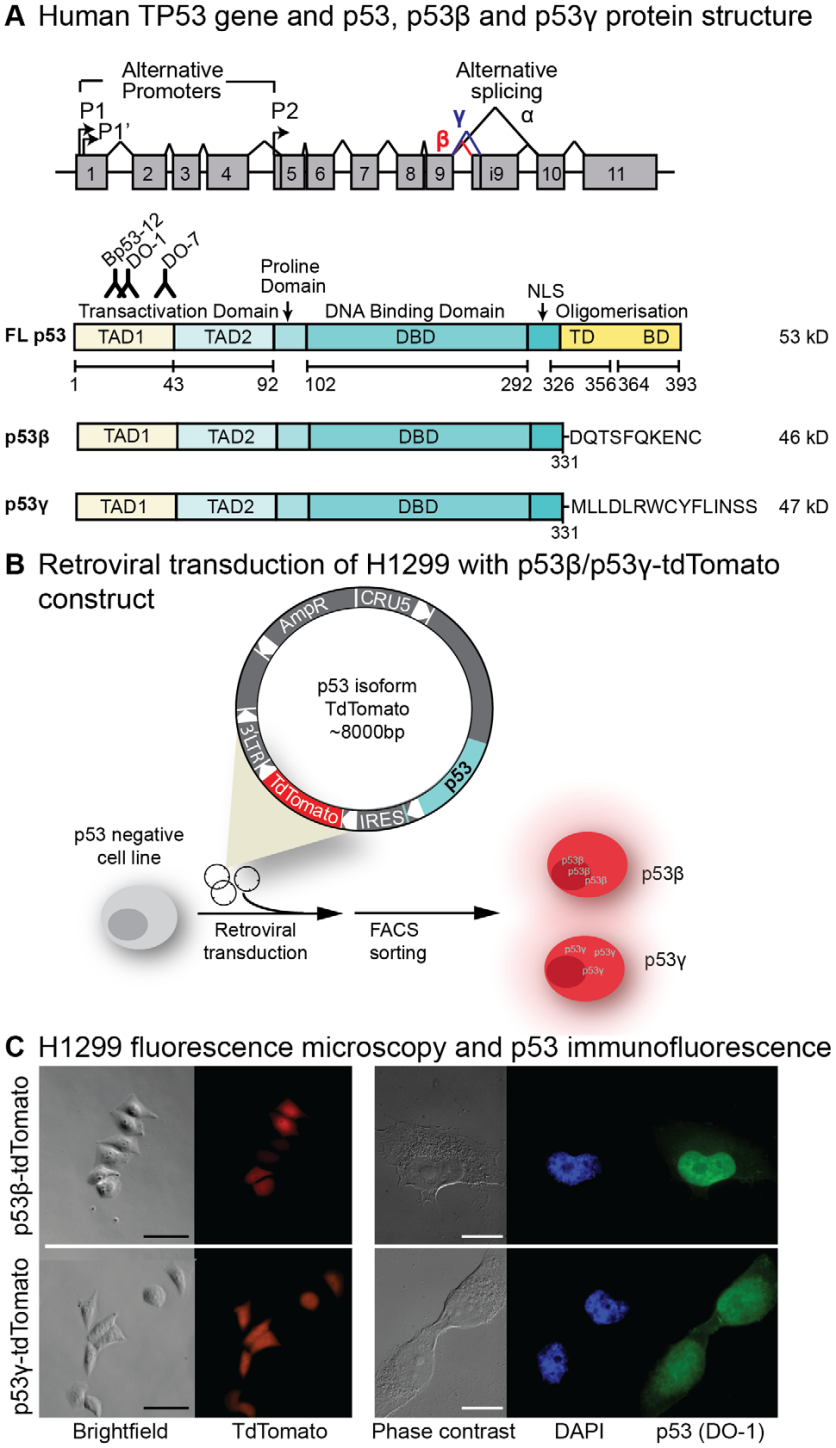
p53 isoforms and experimental setup. (**A**) The human TP53 gene have alternative promoters (P1, P1′, P_2_) and several alternative splicing sites (full-length p53 (α), β, γ) generating p53 isoforms. Alternative splicing of intron 9 leads to expression of the p53 protein isoforms p53β and p53γ with a truncated carboxy-terminal terminating with 10 and 15 additional amino acids, respectively. Binding site of p53 antibodies Bp53-12 (recognizes amino-acid region 16–25), DO-1 (amino-acids 20–25) and DO-7 (amino-acids 37–45) is indicated. NLS: nuclear localization signal. TD: tetramerization domain. BD: basic domain. (**B**) p53^null^ H1299 lung carcinoma cell line were retrovirally transduced with plasmid vector containing p53 isoforms p53β, p53γ or p53FL and a tdTomato reporter. TdTomato expression allows FACS sorting of successfully transduced cells. (**C**) Fluorescence microscopy confirms tdTomato expression (red) of FACS sorted H1299 cells. Scale bar: 100 µm. p53 (DO-1) immunofluorescence staining (green) show mainly nuclear localization of p53β and both nuclear and cytoplasmic localization of p53γ. DAPI (blue) DNA stain visualize the nucleus. Scale bar: 20 µm.

The gene of the tumor suppressor p53 is shown to encode at least 12 different p53 protein isoforms through alternative splicing, promoter and translational initiation (reviewed in [1], [2]) (Figure 1A). The differential expression of several of these isoforms has recently been established in cancer, [3], [4] though their functional role is not fully understood. Their structural characteristics may indicate isoform specific mechanisms. p53β and p53γ lack the oligomerization domain (Figure 1A) that is required for p53 tetramerization and thus influence p53 DNA binding and transcriptional activity. However, p53β has been shown to bind certain p53 promoters and form protein complexes with full-length p53. Furthermore, p53β and p53γ is expressed in a tissue-specific manner, which may suggest diverse tissue-determined functions that may be reflected in cancer [5]. This complicates a simple understanding of p53 function, but may support future use of p53 isoform profiles in prediction of outcome and drug sensitivity in cancers.

We have previously reported that acute myeloid leukemia patients with high expression of p53β and p53γ protein relative to full-length p53 protein respond better to intensive chemotherapy and have a significant longer survival after treatment [6]. Similarly, in chronic lymphocytic leukemia, there is a strong correlation between an accumulation of full-length p53 protein and inferior outcome [7]. In breast cancer, patients with mutated p53 have more than three times increased risk of recurrence and death compared to patients with wild-type p53, but co-expression of p53γ and mutated p53 leads to similar beneficial prognostic outcomes as those expressing wild-type p53 only [8]. p53β is over-expressed in renal cell carcinomas compared to normal tissue and the p53β mRNA level is significantly associated with tumor stage in these cancers [9]. In addition the p53β concentration is associated with poorly differentiated ovarian cancer and in patients with functionally active p53, expression of p53β correlated with worse recurrence-free survival [10]. Furthermore, a frequent loss of p53β and p53γ has been reported in head and neck squamous carcinoma [11]. These studies suggest that p53β and p53γ may influence carcinogenesis and drug-sensitivity in an organ- and ratio-dependent manner, and emphasize the need to discern their individual function and regulation.

The major negative regulator of full-length p53 protein is a family of E3 ubiquitin ligases, including Mdm2, which mark p53 for proteasomal degradation through polyubiquitination of lysines on the carboxy-terminal tail of p53 [12]. These lysines are lost in the carboxy-terminally truncated p53β and p53γ. Recently it has been demonstrated that the expression level of both p53β and p53γ are regulated by the proteasome and although Mdm2 was found to interact with both isoforms, the degradation of both isoforms was independent of Mdm2 [13]. Therefore, p53β and p53γ may be degraded through an alternative pathway [14]. Interestingly, it has been described that p53 may be degraded by the 20S proteasome by default, independently of ubiquitination. Also, the NAD(P)H quinone oxidoreductase NQO1, localized to the S20 proteasome, is able to stabilize full-length p53, thereby protecting it from ubiquitin-independent proteasomal degradation [15].

It is not known if p53β and p53γ direct biological effects alone, or if they require full-length p53 to function [14]. Therefore, we expressed p53β and p53γ individually in a p53^null^ background using the H1299 lung carcinoma and SAOS-2 osteosarcoma cell lines. We compared functional implications of individual expressed isoforms on clonogenicity, examined the proteasomal route of degradation, and tested functional impact on chemosensitivity *in vitro* and tumor growth in a xenograft model. p53β and p53γ were found to have a chemosensitizing effect as well as an increased tumor growth potential *in vivo*.

## Results

### Stable expression of p53β and p53γ in p53^null^ H1299 lung carcinoma cells

**Figure 2:**
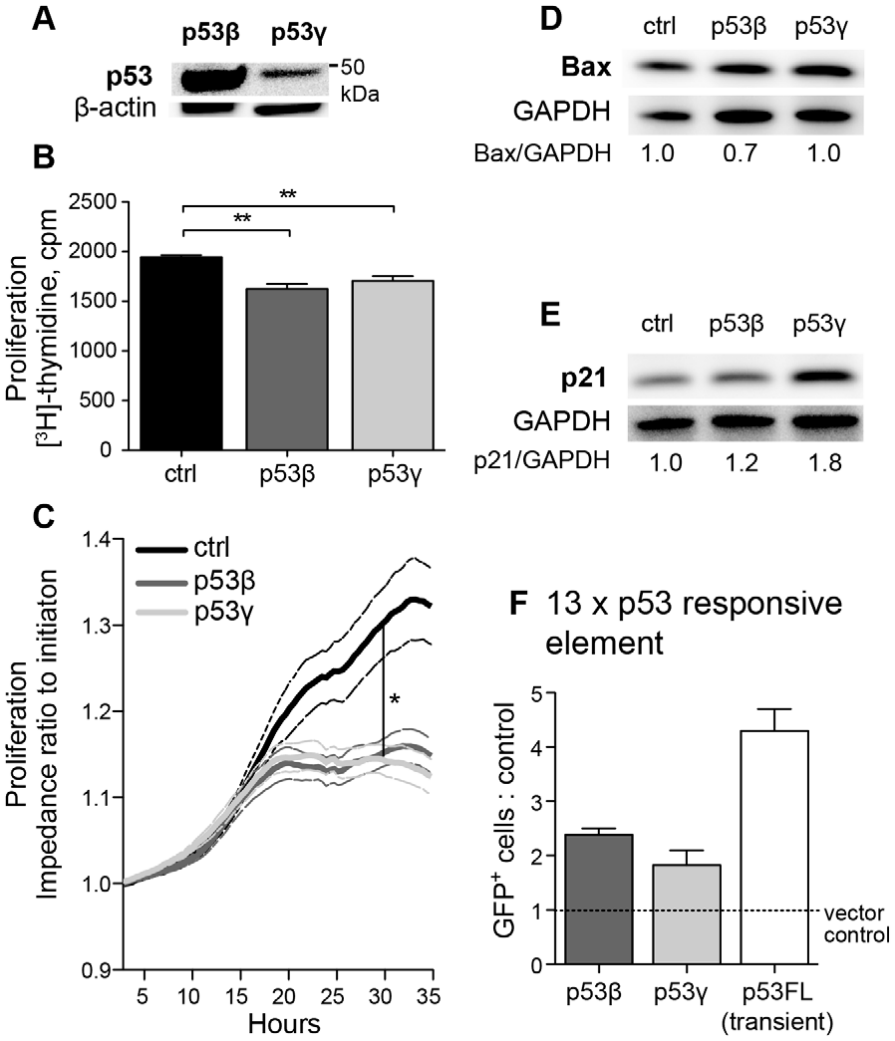
Basal characteristics of H1299 p53β and H1299 p53γ. (**A**) p53 immunoblot (Bp53-12) of H1299 cells transduced with p53β-tdTomato and p53γ-tdTomato construct. β-actin was used as loading control. (**B**) ^3^H-thymidine incorporation assay of H1299 p53β, H1299 p53γ and H1299 control cells (tdTomato vector control). The graph shows results from four individual experiments (average of 18 wells each). The experiments have been normalized. ** *P*-value<0.01. Error bars: Standard Error of the Mean (SEM). (**C**) Cell proliferation measured by Electric-Substrate Impedance Sensing. Impedance values are normalized after initial cell stabilization and shown as ratio of normalization value. The graph shows results from four measurements of vector control cells and six measurements of p53β^+^ and p53γ^+^ cells from two separate experiments. Standard error of mean is denoted by dotted lines. Highest variation in cell proliferation occurred after 30 hours after initiation, *P-value<0.05 calculated by paired Students t-test. (**D**) and (**E**) show immunoblot of basal level of Bax and p21, in both p53β^+^ and p53γ^+^ H1299 cells. GAPDH act as loading control, and the ratio of p21 or Bax to loading control with control value set to 1.0 is indicated. (**F**) Transient transfection of H1299 p53β and H1299 p53γ with the 13×p53RE-GFP construct and H1299 wt cells with both p53FL-construct and 13×p53RE-GFP were analyzed by flow cytometry (n = 2). Results are presented as a ratio of GFP positive cells in H1299 p53β, H1299 p53γ and H1299 cells transiently transfected with full-length p53 to H1299 vector control. Error bars: standard error of mean. Student's t-test give *P*-value 0.053 of p53β cells versus vector control, and *P*-value 0.21 of p53γ versus vector control.

In order to study isoform specific biology, retroviral constructs/vectors containing p53β, p53γ or full-length p53 (Figure 1B) were generated to either retrovirally transduce or transiently transfect p53^null^ cancer cell lines. The p53^null^ lung carcinoma H1299 cell line was retrovirally transduced and FACS-sorted to generate stably expressing p53β, p53γ or full-length p53 H1299 cell lines. Employing a retroviral vector containing the p53 isoform and a fluorescent protein marker (tdTomato), transduction and sorting of tdTomato^+^ cells was performed twice (Figure 1B; see Materials and Methods for experimental details). Sorted cells were evaluated for tdTomato expression by flow cytometry and fluorescence microscopy (Figure 1C), and re-sorted for tdTomato expression if needed. Considerably fewer tdTomato^+^ fluorescent p53γ^+^ cells were observed following all transductions when compared to transduction efficiencies obtained with p53β^+^ cells (not shown). Full-length p53^+^ congenic H1299 cells could not be established, presumably due to the cytotoxic effect of p53 expression. p53 immunofluorescence showed a predominantly nuclear localization of p53β and both nuclear and cytoplasmic localization of p53γ (Figure 1C). p53β or p53γ in H1299 cells was confirmed by PCR of *TP53* segment sequencing (exon 1–12) of both strands and immunoblot of p53 protein isoforms (Figure 2A; for details see Materials and Methods section). Immunoblot showed that p53β was expressed at considerably higher levels compared to p53γ (Figure 2A).

^3^H-thymidine DNA-incorporation was measured to investigate the proliferative capacity of the transduced cells. A small, but significant reduction in proliferation was noted in H1299 p53β and H1299 p53γ compared with H1299 transduction control (*p*<0.01; Figure 2B). The reduced proliferation of both p53β^+^ and p53γ^+^ cells was also perceived by electric cell-substrate impedance sensing (ECIS) when compared to proliferation of vector control cells 30 hours after plating (*p*<0.05; Figure 2C). Performing a colony formation assay of H1299 p53β and H1299 p53γ under hypoxic conditions also demonstrated a reduced tendency of colony formation compared with vector control (mean ± SEM for vector control^+^ cells; 93±13, p53β^+^ cells; 72±4, p53γ^+^ cells; 68±4 (n = 3) (not shown)). Immunoblots illustrated an increased basal-level of p21^(CIP1/WAF1)^ in both H1299 p53β and H1299 p53γ cells, while little change in Bax was noted (Figure 2E, 2D). A p53 promoter reporter assay was performed to determine if p53β or p53γ would be able to activate the genes *DDI2, ARG2, CDKN1A, E2F7, SERPINE1, TP53INP1* or *TP73.* However, no significant activation was detected in untreated or treated (camptothecin, doxorubicin) H1299 p53β or H1299 p53γ cells (not shown). However, a transient transfection of H1299 p53β and H1299 p53γ cells with a 13×p53 responsive element (RE) coupled to a GFP reporter gene, and subsequent analysis for GFP expression by flow cytometry, indicated that p53β and p53γ both may activate this p53 consensus responsive element (Figure 2F).

### Both p53β and p53γ sensitize cells to chemotherapy

**Figure 3:**
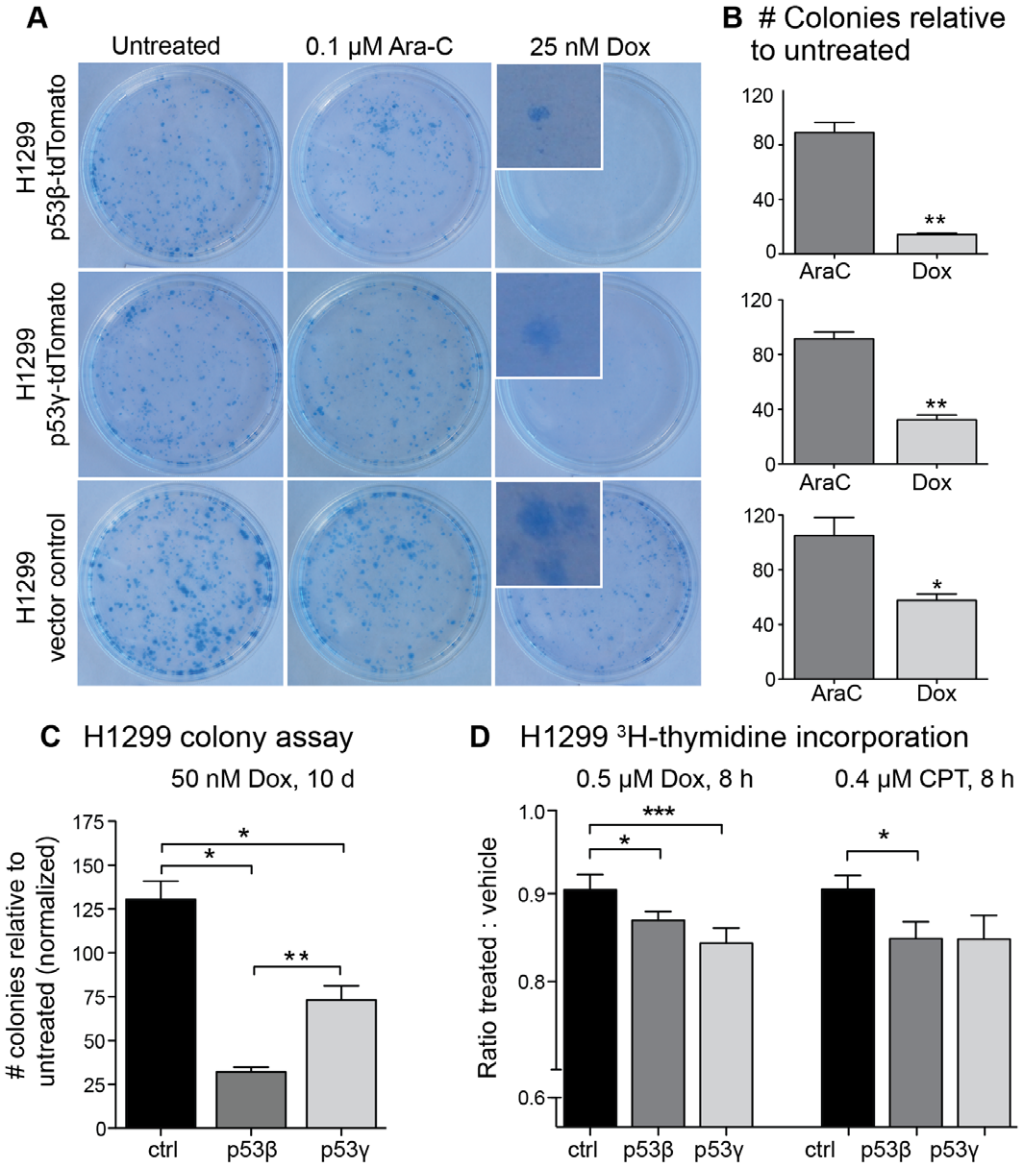
H1299 colony formation and proliferation after chemotherapy. (**A**) Colony formation assay of H1299 p53β, H1299 p53γ and H1299 vector control demonstrated significantly decreased colony formation in p53β^+^ and p53γ^+^ cells after seven-days treatment with 25 nM doxorubicin (Dox). Insert shows a magnification of selected colonies from the doxorubicin treated plates. (**B**) illustrates count of treated colonies relative to colony count of untreated cells from (A). Controls within each group have been normalized to 100 colonies, and the treatment groups have been subsequently adjusted. (**C**) Each bar represents the number of colonies within each treatment group, and statistics have been calculated based on the untreated control within each cell subtype (p53β, p53γ, wt (not transduced) or vector control (ctrl, tdTomato vector control). (**D**) ^3^H-thymidine incorporation assay of p53β^+^ and p53γ^+^ cells exposed for 8 hrs to 0.5 µM Dox (n = 6 experiments), 0.4 µM camptothecin (CPT; n = 2), or vehicle (DMSO) (each separate experiments have six parallels each). Cytarabine (AraC) at 0.1 µM gave no significant response. Columns represent the ratio of treated to control (DMSO) ^3^H-thymidine uptake. * *P*-value<0.05, **P-value<0.01, *** *P*-value<0.001. Error bars: standard error of mean.

Following the observation that the isoforms may activate p53-responsive genes, we examined the response of the H1299 p53β and H1299 p53γ cell lines to chemotherapeutics with a colony-formation assay. The pyrimidine antagonist arabinofuranosyl cytidine (cytarabine, Ara-C), the cytotoxic antibiotic and topoisomerase II inhibitor doxorubicin (Dox) and the topoisomerase I inhibitor camptothecin (CPT) were tested. In both H1299 p53β and H1299 p53γ, a significantly decreased colony formation compared to vector control was observed when treated with doxorubicin (Figure 3A–C). Treatment with Ara-C and camptothecin (not shown) showed less effect on colony formation than doxorubicin, but nevertheless revealed a tendency towards reduced number of colonies. Decreased proliferation of H1299 p53β and H1299 p53γ after treatment with doxorubicin and camptothecin (especially at higher dose) was also identified by a ^3^H-thymidine incorporation assay (Figure 3D). Transient transfection of p53^null^ SAOS-2 osteosarcoma cell line with p53β- and p53γ-tdTomato construct followed by treatment with 0.5 µM doxorubicin for 24 hours, also showed significant reduced ^3^H-thymidine DNA incorporation in p53β^+^ cells (data not shown). This was not seen with the p53γ or full-length p53, and we propose that this lack of doxorubicin toxicity may be caused by high cell death caused by the cell death induction potential of p53γ or full-length p53.

**Figure 4:**
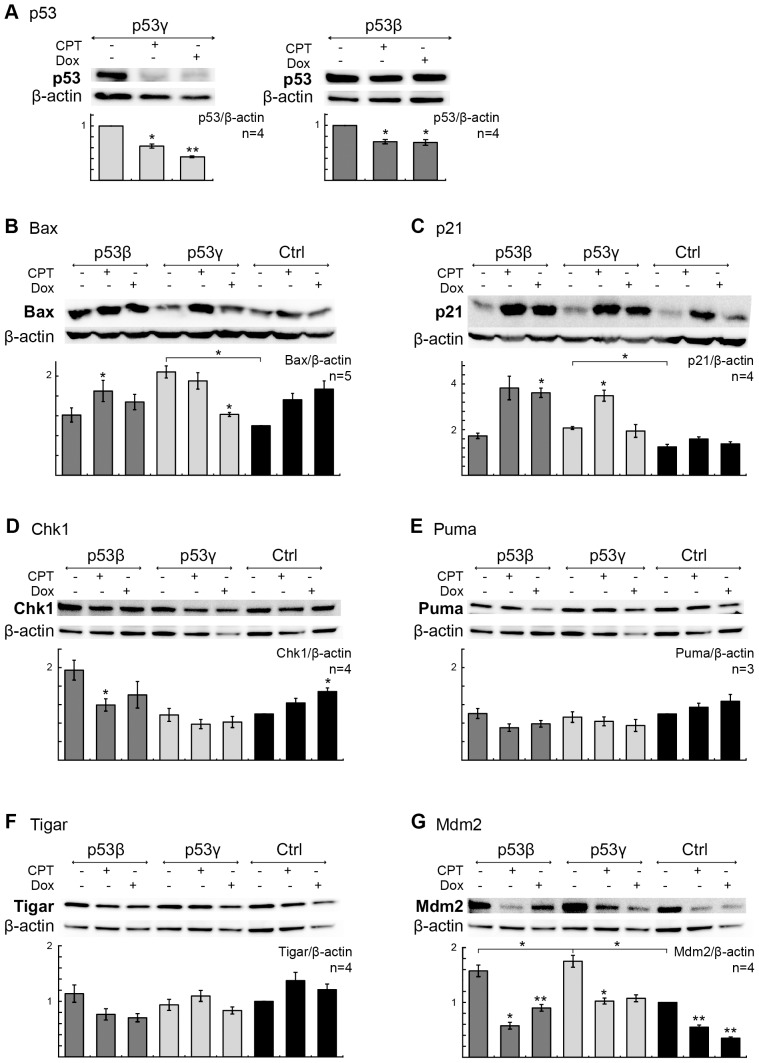
p53β and p53γ protein modulation after treatment with camptothecin or doxorubicin. (**A**) Immunoblot analysis of p53 levels in H1299 p53γ and H1299 p53β cells after treatment with 0.5 µM doxorubicin (Dox) and 0.2 µM camptothecin (CPT) (8 hrs incubation time). Bar graphs represent the mean of four different experiments. * P-value<0.05, ** P-value<0.01. (**B–G**) Immunoblot analysis of Bax, p21, Chk1, Puma, Tigar and Mdm2 levels in H1299 p53β, H1299 p53γ and H1299 vector control cells after treatment with 0.5 µM Dox and 0.2 µM CPT (8 hrs). β-actin act as loading control, and the ratio of p53, Bax, p21,Chk1, Puma, Tigar or Mdm2 to loading control compared to value of untreated vector control cells (set to 1.0) is indicated. Bar graphs represent the mean of five (Bax), four (p21, Chk1, Tigar, Mdm2) or three (Puma) different experiments. Error bars: standard error of mean. Protein levels were compared using Student's t-test. * *P*-value<0.05, ** P-value<0.01. Brackets represent significant changes in basal protein levels relative to untreated H1299 vector control cells.

Immunoblots of camptothecin- and doxorubicin-treated H1299 p53β, H1299 p53γ and H1299 vector control cells showed a significant decrease in p53γ and p53β expression indicating degradation of p53β and p53γ protein (Figure 4A). Camptothecin and doxorubicin treatment in H1299 p53β cells amplified the Bax and p21^(CIP1/WAF1)^ response (Figure 4B,C). p53γ^+^ cells showed a significant decrease in Bax after doxorubicin treatment while p21^(CIP1/WAF1)^ was significantly elevated after camptothecin treatment (Figure 4B,C). Furthermore, an increase in basal level of the kinase Chk1 was detected with p53β. Chk1 was significantly reduced upon camptothecin treatment (Figure 4D) and no clear changes in Puma protein levels were seen between the groups (Figure 4E). Decreased protein levels of Tigar (TP53-induced glycolysis and apoptosis factor) protein in p53β^+^ and p53γ^+^ cells after doxorubicin treatment were observed (Figure 4F). Increased basal Mdm2 levels were observed in both p53β and p53γ, and reduced in all cells after chemotherapy treatments (Figure 4G).

### Proteasomal inhibition increases p53γ but not p53β

**Figure 5:**
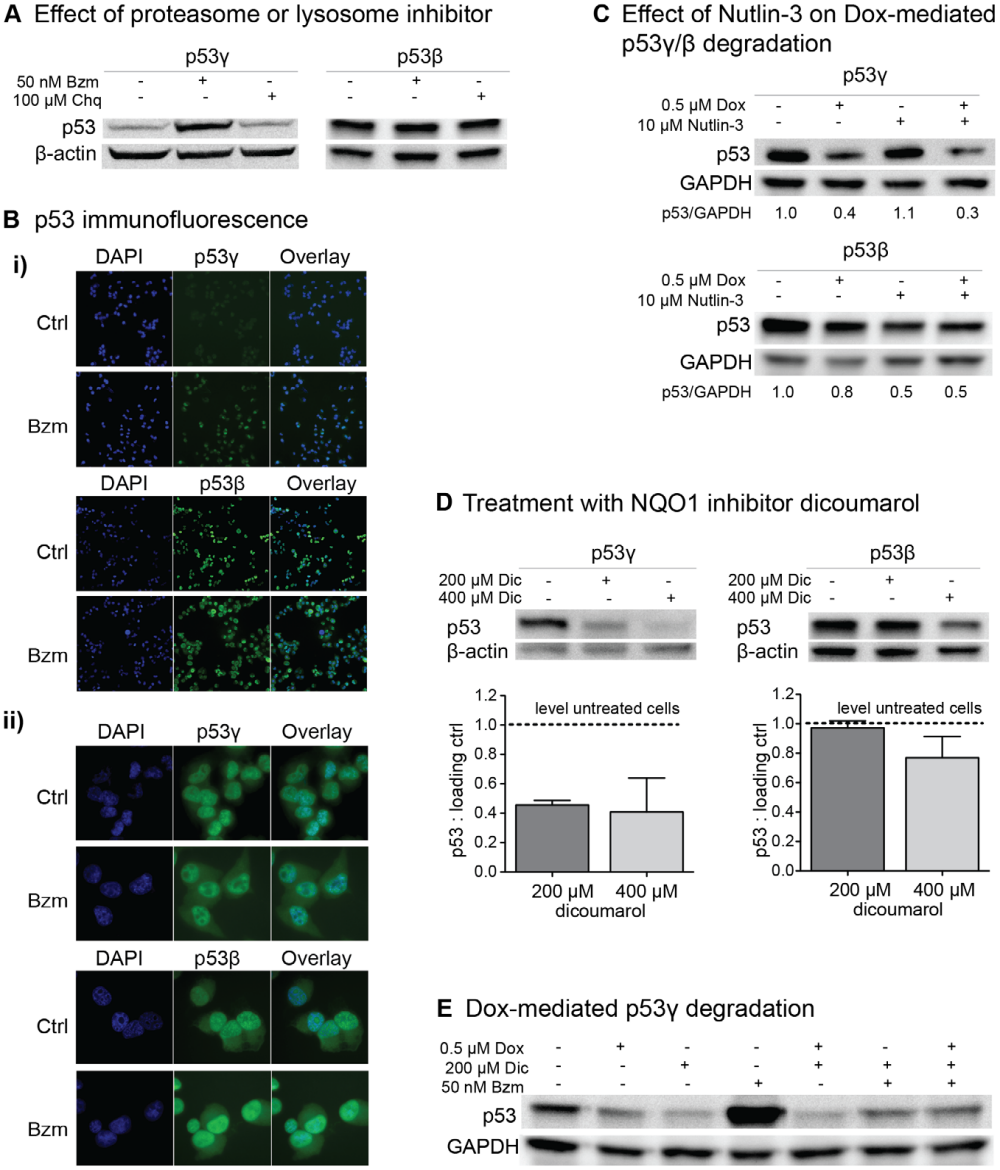
Protein stabilization of p53β and p53γ. (**A**) Immunoblot of p53γ and p53β protein after treatment with 50 nM proteasome inhibitor bortezomib (Bzm) or 100 µM lysosome inhibitor chloroquine (Chq) for 8 hrs. (**B**) Immunofluorescence images of H1299 p53γ and H1299 p53β−/+bortezomib treatment (Bzm; 50 nM, 8 hrs). p53 (DO-1) immunofluorescence staining in green. DAPI (blue): nucleic acid stain. i) shows the differences in expression levels (10× magnification), while ii) enlightens the subcellular localization (63× magnification). (**C**) Treatment of H1299 p53γ (top) and H1299 p53β (bottom) with doxorubicin (0.5 µM) and nutlin-3 (10 µM) for 8 hrs. GAPDH is included as a loading control. The ratio of p53 to loading control compared to the untreated vector control cells (set to 1.0) is indicated. (**D**) Treatment of H1299 p53γ (left) and H1299 p53β (right) with NQO1-inhibitor dicoumarol for 8 hrs. Quantification of protein signal presented as ratio of p53 to β-actin in lower panel (n = 2 immunoblots). β-actin was included as a loading control. Error bars: standard error of mean. (**E**) Protein levels of p53γ and Mdm2 after treatment of H1299 p53γ with 0.5 µM doxorubicin (Dox), 200 µM dicoumarol (Dic) and 50 nM bortezomib (Bzm) for 8 hours (a representative immunoblot shown). GAPDH is included as a loading control.

The p53γ protein was considerably degraded after doxorubicin exposure (Figure 4A). To investigate the mechanism of degradation, H1299 p53β and H1299 p53γ cells were treated with either the proteasome inhibitor bortezomib (Bzm) or the lysosome inhibitor chloroquine (Chq). Immunoblotting demonstrated that while p53γ levels were elevated considerably subsequent to treatment with bortezomib, p53β displayed stable protein levels after bortezomib and chloroquine treatment (Figure 5A). These findings was further confirmed at the subcellular level through immunofluorescence of the H1299 p53β and H1299 p53γ cells (Figure 5B) where an increase in fluorescence was observed for p53γ after 8 hours of bortezomib treatment while the level of p53β appeared unaffected (Figure 5B (i)). An incubation time of 8 hours with bortezomib was needed in order to inhibit p53γ degradation since treatment for 1 hour, 2 hours and 4.5 hours did not result in an inhibition of p53γ degradation and also no difference in the expression level of p53β was observed using these time points (data not shown). To further investigate the subcellular localization of p53γ and p53β after bortezomib treatment as compared to untreated cells, the cells was investigated using a higher magnification where the untreated p53γ cells were overexposed in order to capture the localization (Figure 5B ii)). It is evident in both untreated and bortezomib treated cells that p53γ is concentrated in the nucleus, excluded from the nucleoli and only observed diffusely in the cytoplasm. Without treatment, the p53β protein was concentrated in the nucleus, localized in the nucleoli and in a speckled nucleoplasmic pattern, in addition to diffusely in the cytoplasm. After bortezomib treatment the nuclear localization of p53β appeared more diffuse. No change in p53γ and p53β stability or subcellular localization was observed after exposure to chloroquine at the time points described above (data not shown).

To investigate if Mdm2 could have a role in degradation of p53β or p53γ, the H1299 cells were co-treated with the p53-Mdm2 inhibitor nutlin-3 in addition to doxorubicin (Figure 5C). The nutlin-3 treatment alone had no effect on p53γ stability and the doxorubicin-induced degradation of p53γ was not rescued by treatment with nutlin-3, indicating a Mdm2 independent proteasomal degradation of p53γ (Figure 5C). The p53β protein, on the other hand, was to a higher degree degraded after treatment with nutlin-3 compared to doxorubicin, indicating that Mdm2 is involved in p53β stability (Figure 5C). To investigate whether p53γ could be regulated by NQO1, as reported for full-length p53, p53γ^+^- cells were treated with the NQO1-inhibitor dicoumarol. A dose dependent degradation of p53γ after dicoumarol exposure demonstrates that NQO1 is indeed involved in the regulation of p53γ. p53β was less affected by dicoumarol-treatment and only the high-dose treatment resulted in a minor reduction in the p53β protein level (Figure 5D). In order to further map the degradation pathway of p53γ after doxorubicin treatment, p53γ^+^ cells were treated with doxorubicin in combination with dicoumarol, which resulted in a further degradation of p53γ (Figure 5E). This degradation was partly rescued by bortezomib treatment.

### Growth advantage of p53β and p53γ expressing H1299 cells *in vivo* but not *in vitro*

**Figure 6:**
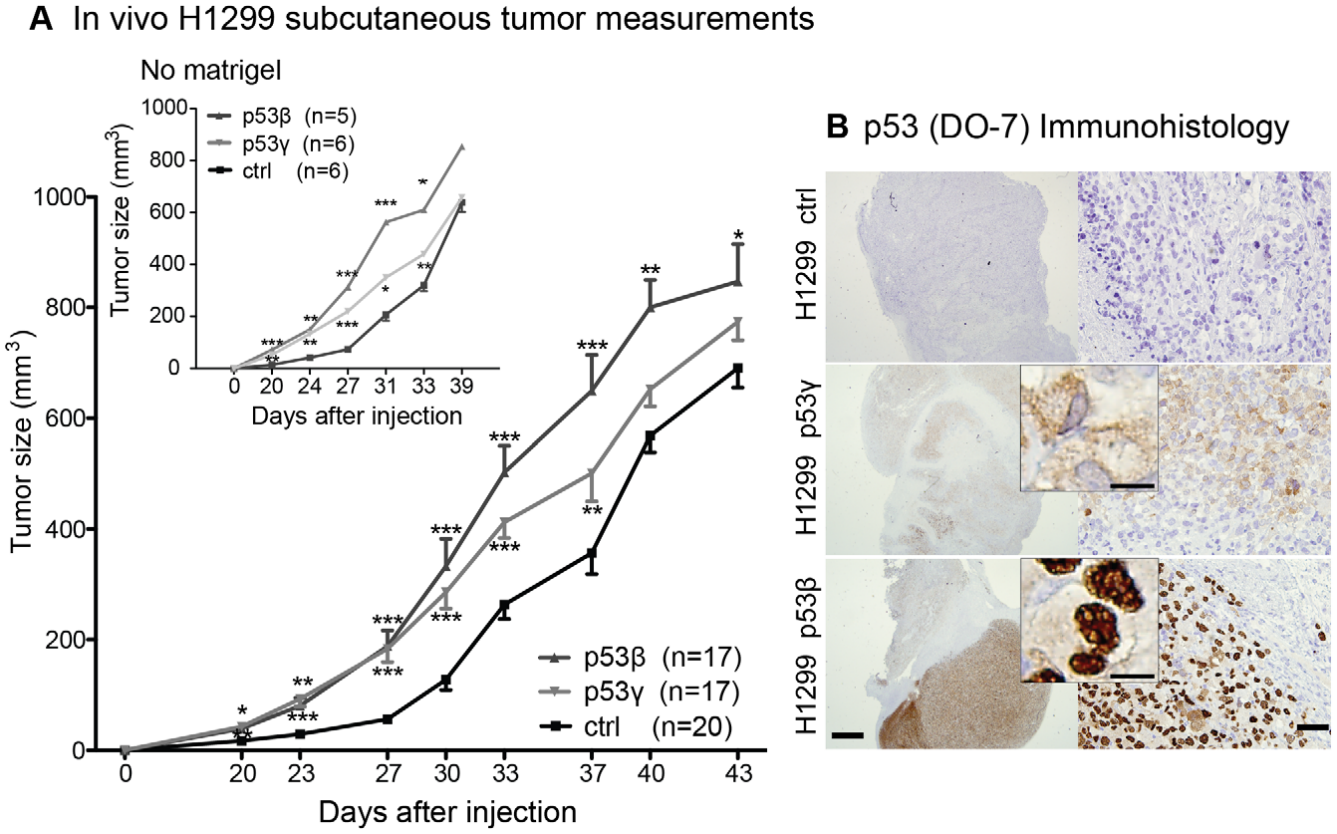
Tumor growth of H1299 p53β and H1299 p53γ cells. (**A**) H1299 *in vivo* s.c. tumors were measured in 3 independent experiments. In total: p53β^+^ tumors n = 17; p53γ^+^ tumors n = 17; Control vector (Ctrl; tdTomato^+^) n = 20. The tumor sizes of p53β^+^ and p53γ^+^ tumors were compared to ctrl tumors using Students t-test. * *P*-value<0.05, ** *P*-value<0.001, *** *P*-value<0.0001. Insert (top left corner) shows tumor measurements of tumors that were injected without matrigel. Error bars: standard error of mean. (**B**) Immunohistochemistry of p53 (DO-7) of s.c. tumors. Top: p53-negative vector control tumor (Ctrl). Middle: p53γ^+^ tumor. Bottom: p53β^+^ tumor. Scale bar left images 1 mm, right images 50 µm, middle insert 10 µm.

To examine the function of p53β and p53γ protein expression in a more realistic cancer environment exhibiting hypoxia and low nutrition, we examined the growth of subcutaneous H1299 in NSG mice. Surprisingly, an early tumor growth initiation followed by a significantly increased tumor growth was found of both p53γ and p53β congenic H1299 cells, compared to vector control (Figure 6A). This was in contrast to *in vitro* findings whereby the ^3^H-thymidine-incorporation assay showed only a minor decrease in proliferation of p53β^+^ and p53γ^+^ cells (Figure 2B). To evaluate if the growth factors present in the matrigel (for example TGF-β, epidermal growth factor, insulin-like growth factor and fibroblast growth factor) injected with the tumor cells could influence growth of p53β^+^ and p53γ^+^ tumors, the same assay was performed without matrigel. However, the same pattern was observed with these tumors (Figure 6A, top left graph). Immunohistochemistry of p53 in tumors (Figure 6B) reflected the previous *in vitro* observations by immunofluorescence (Figure 1C) demonstrating strong p53β signals (bottom images) with a major localization to the nucleus (insert), and p53γ signals of predominantly cytoplasmic origins.

## Discussion

Stably transduced H1299 cells demonstrated enhanced chemosensitivity to doxorubicin and camptothecin after introduction of p53β and p53γ (Figures 3, 4). This was particularly evident in the colony formation assays, which reflects the total sum of all proliferative, differentiation, senescence and cell death effects [16]. Immunoblot analysis demonstrated an upregulation of p21^(CIP1/WAF1)^ and Bax after exposure to doxorubicin and camptothecin, apparently through a p53-independent mechanism, but with an enhanced p21^(CIP1/WAF1)^ response in p53β^+^ and p53γ^+^ cells especially in response to camptothecin. It has previously been reported that p53β may bind to the p21^(CIP1/WAF1)^ promoter sequences [5]. p21^(CIP1/WAF1)^ promotes cellular arrest, but may also promote apoptosis through both p53-dependent and independent mechanisms under certain cellular stress (reviewed in [17]), dependent upon upregulation of pro-apoptotic Bax [18]. This may also explain the reduced ^3^H-thymidine incorporation observed in CPT-treated p53β cells. We found that p53β and p53γ may have an effect on an optimized p53-responsive element (Fig. 2F), but a direct activation of p21^(CIP1/WAF1)^ promoter assay was only indicated and not significant (data not shown). However, the basal level of p21^(CIP1/WAF1)^ protein was found to be increased by p53β and p53γ expression (Figure 2E). Furthermore, the basal levels of Mdm2 were also found to be increased by p53β and p53γ expression (Figure 4G). Although a p53 promoter reporter assay did not detect activation of the genes tested (data not shown, see Materials and Methods section for details), a weak positive signal by a 13×p53 responsive element reporter was observed (Figure 2E). Thus, we cannot rule out p53β and p53γ modulation of these p53 targeted genes at the posttranslational level or a weak gene induction not detected by the promoter reporter assay.

We find that resting cells stably transduced with p53γ only express low levels of p53 isoform protein, corresponding with previous reports that p53γ may be cytotoxic [19]. We propose that this low level is sufficient to induce muted levels of p21^(CIP1/WAF1)^ protein and is accompanied by a tendency towards decreased proliferation and clonogenicity *in vitro*. Further experiments are needed to determine if the isoforms respond specifically to different chemotherapeutics.

Protein levels of p53β and p53γ decreased after treatment with camptothecin or doxorubicin, and p53γ was particularly attenuated following therapy with doxorubicin. Through treatment with proteasome-, lysosome- and a NQO1-inhibitor, we suggest that the stability of p53β and p53γ are differentially regulated (Figure 5). Treatment with nutlin-3, a Mdm2-binding inhibitor of the Mdm2-p53 interaction, did not result in an increased level of p53β or p53γ, suggesting that Mdm2 is not a negative regulator of p53β/γ and consistent with previous reports [13], [19]. However, our experiments indicate that p53γ is degraded by the proteasome (Figure 5). Conflicting reports exist on proteasomal degradation of p53β and p53γ [13] [20]. It is also reported that Mdm2 interact with both isoforms, but only promote ubiquitination of p53β. However, Mdm2-promoted stabilization of p53β is suggested through neddylation [13]. This could explain the decreased level of p53β we observed after treatment with nutlin-3 (Figure 5C). It was recently suggested that p53 may be proteasomally degraded by default in an Mdm2 and ubiquitin-independent manner, and that p53 is stabilized by the NAD(P)H quinone oxidoreductase NQO1 [15]. Treatment of H1299 p53γ and p53β cells with the NQO1 inhibitor dicuomarol (Figure 5) resulted in a dose dependent degradation of p53γ but not p53β, suggesting that NQO1 may be an important enzyme in proteasomal processing of p53γ. This was further confirmed with an increased degradation of p53γ after combining doxorubicin and dicuomarol treatment (Figure 5E). Together, these observations emphasize the importance to further characterize the route of degradation of p53β and p53γ.

p53 is proposed to play a role in metabolic regulation of tumor growth, including tumor responses to hypoxia and nutritional deprivation [21], [22]. Therefore, we compared tumor growth in a subcutaneous xenograft. Both H1299 p53γ and H1299 p53β lead to significantly increased size of tumors compared to vector control cells. A critical stage of early tumor progression is an adaptation to hypoxic and acidic conditions and a change to aerobic glycolysis to promote further tumor expansion [23], [24]. Thus, the finding that p53β and p53γ cells caused a significantly earlier initiation of tumor growth indicates that they play a role in the adaptive response to metabolic stress. It has recently been suggested that p53 isoforms may have more specific metabolic functions that promote metabolic adaptation [21], and tumor growth advantage in serum-nutrient starvation is suggested through modulation of p21^(CIP1/WAF1)^ [25]. Engraftment without the use of matrigel resulted in the same tumor size profile, eliminating interference by the matrigel basement membrane matrix injected with the cells (Figure 6, insert). Further investigation of the role of the various isoforms and their response to metabolic stress may contribute to additional understanding of their role in cancer.

In summary, we suggest that p53β and p53γ individually imply functional effects in cancer cell lines. Future studies are needed to investigate if the function of p53β and p53γ at defined expression levels, and to delineate the mechanisms of p53 isoform regulation in cancer growth and chemotherapy.

## Materials and Methods

### Cell culture and reagents

NCI-H1299 non-small cell lung carcinoma cell line (DSMZ, The German Resource Centre for Biological Material, Braunschweig, Germany) were maintained in Roswell Park Memorial Institute (RPMI)-1640 medium (Sigma-Aldrich, Inc. St. Louis, MO, USA) and SAOS-2 osteosarcoma cell line (DSMZ) was cultured in McCoy's 5A medium (Sigma-Aldrich) supplemented with 10% and 15%, respectively, of heat-inactivated Fetal Bovine Serum (FBS) (PAA Laboratories GmbH, Pasching, Austria), 1% penicillin/streptomycin (PS) (Sigma-Aldrich) and 1% L-glutamine (Sigma-Aldrich) and the cells were incubated in a 5% humidified atmosphere at 37°C. Cells were treated with camptothecin (Sigma-Aldrich), doxorubicin (Pfizer Inc., New York, NY, USA), arabinofuranosyl cytidine (Sigma-Aldrich), dicoumarol (Sigma-Aldrich), nutlin-3 (Cayman Chemical Company, Ann Arbor, MI, USA), bortezomib (Millenium Pharmaceuticals, Cambridge, MA, USA) and chloroquine (Sigma-Aldrich), as indicated in the text.

### Design of p53isoform-tdTomato constructs

Expression vectors for p53 isoforms p53FL, p53β, and p53γ were generously provided by Dr. Bourdon (University of Dundee, Scotland, UK). The p53 segments from these plasmids were cloned into an MMLV retroviral vector (L335, D.R. Micklem, unpublished) upstream of an IRES-tdTomato reporter gene. This vector drives constitutive transcription of a bicistronic mRNA comprising the cloned gene followed by an internal ribosome entry site and the red fluorescent protein tdTomato. The predicted sequence of the vector is available upon request. p53 isoforms were amplified by PCR using a forward primer containing EcoRI and SfiI sites (at gaa ttc ggc cat tac ggc cac acc ATG GAG GAG CCG CAG TCA GAT) and reverse primers containing BamHI and SfiI sites (p53FL: ta gga tcc ggc cga ggc ggc cat ata TCA GTC TGA GTC AGG CCC TTC; p53β: ga gga tcc ggc cga ggc ggc cat cta AGG CAA AGT CAT AGA ACC ATT; p53γ: gc gga tcc ggc cga ggc ggc cga ata CAC GGA TAA TAT TTT CAA CTT; overlap with the p53 gene in capitals). After digestion with SfiI, the p53 isoforms were cloned into matching SfiI sites upstream of the IRES-tdTomato reporter gene. Correct inserts were confirmed by sequencing.

### Retroviral transduction of NCI-H1299 cells

NCI-H1299 (p53^−/−^) cells made to stably express p53β, p53γ and control by retroviral transduction with the p53β-tdTomato vector, p53γ-tdTomato vector, and tdTomato only vector (transfection control). Production of infectious retroviral vector particles in 293-based Phoenix A packaging cells and infection of cells were carried out as described [26].

### Transient transfection of cells

H1299 (2×10^3^) or SAOS-2 cells (7.5×10^3^) were seeded in 96-well plates and transfected with the p53 isoform -tdTomato constructs or 13×p53RE-GFP reporter plasmid [27], [28] (a generous gift from Professor M Laiho, University of Helsinki, Finland) using FuGENE 6 Transfection Reagent (Roche Diagnostics, GMbH, Mannheim, Germany) according to manufacturers instructions.

### Sequencing

The p53 isoform- tdTomato construct were sequenced both prior to transduction and after transduction into H1299 cells to confirm correct TP53 isoform sequence. DNA was purified from the cells using DNeasy Blood and Tissue Kit (Qiagen Inc., Valencia, CA, USA), and the concentration was calculated by NanoDrop UV-Vis Spectrophotometer (Thermo Scientific, Wilmington, DE, USA). By PCR using a forward primer for p53 (5′- 3′) a reverse primer for p53β (5′- 3′) and a reverse primer for p53γ (5′- 3′) [5] the p53 isoform product were amplified. This product was characterized by agarose gel separation to confirm segment, and purified using ExoSAP-IT according to suppliers instructions (USB Corporation, Cleveland, Ohio USA). Sequencing PCR was performed using the abovementioned primers in addition to primers towards the middle of the p53 sequence to make sure that the sequencing reaction detects the whole segment: p53 forward primer (5′ gg ccc atc ctc acc atc atc-3′) and reverse primer (5′-c agg gga gta cgt gca agt-3′), with the BigDye Terminator v1.1 Cycle sequencing Kit (Applied Biosystems, Foster City, CA, USA). Sequences were analyzed using DNA sequencing chromatogram trace viewer FinchTV v1.4.0 (Geospiza Inc., Seattle, WA, USA) and EMBOSS Pairwise Sequence Alignment Matcher.

### Colony Formation Assay

A total of 500 cells were seeded per 10 cm^2^ dish in 8 ml of RPMI supplemented with 10% FBS, 1% penicillin/streptomycin and 1% L-glutamine. Treatment with 5 nM camptothecin, 25 nM doxorubicin, and 0.1 µM arabinofuranosyl cytidine (cytarabine, Ara-C) was initiated following 3 days of culture, and the assay terminated at day 10. Colonies were washed twice in 1×PBS before 3 ml of 95% methanol was added for 3 minutes, washed in 1×PBS and stained with 1∶5 solution of tryphan blue stain solution (Thermo Fisher Scientific, Hanover Park, IL, USA) in ddH_2_O. Colony assay was also performed at hypoxic conditions with 1.5% O_2_.

### Immunoblot analysis

NCI-H1299 cells were lysed according to Shieh et al [29] before analysis with one-dimensional gel electrophoresis, according to standard procedures described in [30]. p53 protein was detected using Bp53-12 antibody (Santa Cruz Biotechnology Inc., Santa Cruz, CA, USA), p21^(CIP1/WAF1)^ protein by EA10 p21 antibody (ab16767, Abcam, Cambridge, UK), Bax by Bax 2D2 antibody (sc-20067, Santa Cruz Biotechnology), Chk1 by Chk1 2G11D5 antibody (sc-56288, Santa Cruz Biotechnology), Puma by a polyclonal Puma Antibody (#4976, Cell Signaling Technology, Inc., Danvers, MA, USA), Tigar by Tigar antibody Woody-1 (ALX-804-872C100, Enzo Life Sciences Inc., Farmingdale, NY, USA), Mdm2 by Mdm2 antibody Ab-2 (2A10) (OP115, Merck Millipore, Darmstadt, Germany), GAPDH by GAPDH mAbcam 9484 (Abcam) and β-actin was detected by anti-β-actin, sc-47778 (Santa Cruz Biotechnology). Primary antibodies were followed by secondary horseradish peroxidase conjugated mouse or rabbit antibodies (Jackson ImmunoResearch, West Grove, PA, USA). Membranes were visualized using Pico Stable peroxide solution and luminol enhancer solution (Pierce Biotechnology, Inc., Rockford, IL, USA). Protein bands were detected by Kodak Image Station 4000R (Eastman Kodak Company, Rochester, NY, USA), and quantified using the Carestream MI (Carestream Molecular Imaging, Woodbridge, CT, USA) analysis software. Data were exported to Excel spreadsheet and corrected for background and loading control signal, prior to statistical analysis in the Graphpad PRISM software, using the paired Student's *t*-test to compare two and two groups.

### ^3^H-thymidine DNA incorporation

H1299 cells (2×10^3^ or 5×10^3^) were seeded in 96 well plates and left to settle for 20 hours prior to treatment. The cells were treated for 8 or 12 hrs, and ^3^H -thymidine (1 mCi per well; TRA310, Amersham International, Amersham, UK) was added the last 6 or 10 hours of the treatment period, respectively. For basal proliferation experiments the cells were incubated with ^3^H-thymidine for 10 hours. Cells were harvested and DNA synthesis was determined by ^3^H-thymidine incorporation assays as described [31]. Statistical analysis was performed using GraphPad PRISM (version 5.0b, GraphPad Software, Inc., La Jolla, CA, USA) software. Groups were compared using paired Student's *t*-test.

### Electric-Substrate Impedance Sensing cell proliferation assay

Growth potential of the H1299 cells were assessed using by Electric-Substrate Impedance Sensing (ECIS; Applied Biophysics, Troy, NY) [32], [33]. 2×10^4^ cells were seeded at 5×10^4^/ml concentration in 8W10E+ plates coated with cysteine, and cultured in RPMI supplemented with 10% FBS. Impedance was measured at 64 kHz every 30 seconds for two days.

### p53 Immunofluorescence

2×10^5^ H1299 p53β, H1299 p53γ and H1299 control cells (tdTomato only) were grown on coverslips immersed in 0.5 ml RPMI medium with 10% FBS and 1% L-glutamine. The cells were fixed and permeabilized with 4% paraformaldehyde and ice-cold 99% methanol, respectively, before blocking with 0.5% BSA (Roche Diagnostics GmbH) in 1×PBS. Next, cells were treated with primary p53 antibody (1∶100 or 1∶50, mouse anti-human p53, cat. 554293 BD Pharmingen) diluted in 1×PBS with 0.5% BSA and incubated at 4°C over night before incubation with secondary antibody (1∶5,000 of Alexa 488 goat anti-mouse (Invitrogen Molecular Probes)) diluted in 1×PBS with 0.5% BSA, and incubated in the dark for 1 hour at room temperature. Last, the coverslip was washed 1×PBS and mounted in 5 µl Fluoro-gel II with DAPI (Electron Microscopy Sciences, PA, USA). Images of cell fluorescence were acquired with a Zeiss Axio Observer Z1 inverted microscope (Carl Zeiss Microimaging GmbH, Germany) and analyzed by the AxioVision 4.8.2 software.

### Flow cytometric analysis

1–5×10^6^ transduced H1299 cells were washed twice in 1×PBS and suspended in 1×PBS at a concentration of 5×10^6^ cells/ml. Stably expressing tdTomato^+^ cells were isolated by a Fluorescence Activated Cell Sorter (FACSAria, BD Biosciences) using a 532 nm laser. TdTomato expression was regularly evaluated on an Accuri (Accuri Cytometers Ltd., St. Ives, Cambs UK) flow cytometer to keep TdTomato expression equal between the three H1299 subclones, and cells were re-sorted if needed.

### Luciferase reporter assay

A p53 reporter assay, p53 Biomarker Set (genes *DDI2, ARG2, CDKN1A, E2F7, SERPINE1, TP53INP1* or *TP73*) (SwitchGear Genomics, CA, USA), was transiently transfected into H1299 p53β, H1299 p53γ and H1299 transduction control cells using FuGENE 6 (Roche Diagnostics, GMbH, Mannheim, Germany) according to manufacturers instructions. In a 96-well format, 2500 cells were seeded into each well and transfected after 24 hrs incubation using 0.05 µg of each individual construct. Treatments (camptothecin, doxorubicin) or vehicle control was added to the wells 12 hrs after transfection. After additional 24 hrs incubation Steady-Glo (Promega, WI, USA) was added to the wells and luminescence was measured in an Infinite M200 luminometer (TECAN, Männedorf, Switzerland).

### General animal care and ethics statement

All experiments were approved by The Norwegian Animal Research Authority under study permit number 2008 070, and conducted according to The European Convention for the Protection of Vertebrates Used for Scientific Purposes. Mice capable of engrafting human cancer cell lines NOD/LtSz-Prkdc^scid^/IL2Rγ^null^ mice (abbreviated as NSG) [34], [35] originally from Dr. Leonard Schultz, Jackson Laboratories, Bar Harbour, ME, USA) were housed in groups of five or less in individually ventilated cages (Techniplast, Buguggiate, Italy) and kept on a 12 hrs dark/light schedule at a constant temperature of 21°C and 50% relative humidity. The mice had continuous access to food and autoclaved water.

### Subcutaneous tumor model

5×10^6^ NCI-H1299 p53β-tdTomato, NCI-H1299 p53γ-tdTomato, NCI-H1299 tdTomato and NCI-H1299 wt cells were suspended in 100 µl sterile 1×PBS with 10% Matrigel (BD Matrigel™ Basement Membrane Matrix, BD Biosciences) for s.c. inoculation, and injected with a 28 G syringe. Animals were monitored closely for tumor growth, and tumor volume was measured twice weekly using digital calipers. The mice were euthanized when tumor size reached 1000 mm^3^.

### Histology

Tumor samples collected following euthanasia were transferred to a tube containing 4% formalin for paraffin embedding, cryosectioning and subsequent immunohistochemistry of the samples. Sections were stained with hematoxylin and eosin (H&E) and p53 (DO-7, Dako) (appearing as brown stain). Results were analyzed by standard light microscopy (Olympus BX51, Olympus Corp., Tokyo 163-0914, Japan).
